# Emergency medical care of incarcerated patients: Opportunities for improvement and cost savings

**DOI:** 10.1371/journal.pone.0232243

**Published:** 2020-04-27

**Authors:** Rebecca A. Martin, Rosanna Couture, Nicole Tasker, Christine Carter, David M. Copeland, Mary Kibler, Jessica S. Whittle

**Affiliations:** 1 Department of Emergency Medicine, The University of Tennessee Health Science Center College of Medicine at Chattanooga, Chattanooga, Tennessee, United States of America; 2 Erlanger Health System, Chattanooga, Tennessee, United States of America; 3 The University of Tennessee College of Medicine, Memphis, Tennessee, United States of America; Hofstra University, UNITED STATES

## Abstract

In the United States (US), the lifetime incidence of incarceration is 6.6%, exceeding that of any other nation. Compared to the general US population, incarcerated individuals are disproportionally affected by chronic health conditions, mental illness, and substance use disorders. Barriers to accessing medical care are common in correctional facilities. We sought to characterize the local incarcerated patient population and explore barriers to medical care in these patients. We conducted a retrospective, observational cohort study by reviewing the medical records of incarcerated patients presenting to the adult emergency department (ED) of a single academic, tertiary care facility with medical or psychiatric (med/psych) and trauma-related emergencies between January 2012 and December 2014. Data on demographics, medical complexity, trauma intentionality, and barriers to medical care were analyzed using descriptive statistics, unpaired student’s t-test or one-way analysis of variance for continuous variables, and chi-square analysis or Fisher’s exact test as appropriate. Trauma patients were younger with fewer medical comorbidities and were less likely to be admitted to the hospital than med/psych patients. 47.8% of injuries resulted from violence or were self-inflicted. Most trauma-related complaints were managed by the emergency medicine physician in the ED. While barriers to medical care were not correlated with hospital admission, 5.4% of med/psych and 2.9% of trauma patients reported barriers as a contributing factor to the ED encounter. Med/psych patients commonly reported a lack of access to medications, while trauma patients reported a delay in medical care. Trauma-related presentations were less medically complex than med/psych-related complaints. Medical management of most injuries required no hospital resources outside of the ED, indicating a potential role for outpatient management of trauma-related complaints. Additional opportunities for health care improvement and cost savings include the implementation of programs that target violence, prevent injuries, and promote the continuity of medical care while incarcerated.

## Introduction

The United States (US) correctional system currently imprisons an estimated 2.4 million people. While it is difficult to compare self-reported data, the US incarceration rate of 693 per 100,000 individuals ranks among the highest in the world [1–3]. Incarcerated persons include those confined to either jail or prison. Prisons are typically defined as facilities under federal or state control where inmates’ sentences are greater than one year duration [4]. In contrast, jails are locally governed facilities that predominantly serve as detainment centers for individuals awaiting trial, parole violators, and nonviolent offenders sentenced to less than one year [4–6]. Because of high turnover in jails, the number of individuals confined to jails each year far exceeds the total US inmate population at any given time [1] For example, an estimated 12 million people entered the local jail system in 2013, averaging 23 days in custody [7].

Racial minorities, the impoverished, and the medically underserved disproportionately comprise the incarcerated population [4,5,8]. Compared to the general US population, incarcerated individuals sustain higher rates of trauma and are more likely to be diagnosed with chronic medical conditions, psychiatric illnesses, substance use disorders, and more advanced medical comorbidities [4,5,8]. Despite a constitutional mandate to provide medical care to incarcerated persons, the correctional system lacks a standardized healthcare system [4,5]. Barriers to accessing health care, such as unperformed medical examinations and unavailable prescription medications, are common [4,5] and may be more prevalent in jails than in federal or state prisons [9].

In contrast to the US incarceration rate, there is a relative lack of research evaluating persons in correctional facilities [10]. Conventional studies often exclude this population because of research regulations, the inherent transience of many members, and access difficulties [10,11]. In our ED, we regularly evaluate incarcerated individuals presenting with medical, psychiatric, and trauma-related emergencies. Because our jurisdiction lacks a large local prison and our hospital is not contracted with a prison, most incarcerated patients present from local jails [5]. To gain a better understanding of the demographics, comorbid conditions, and presenting chief complaints of this population, we conducted a three-year retrospective study of incarcerated patients presenting to the adult emergency department (ED) of a single academic, tertiary care facility. We suspected that a disproportionate number of emergencies would be trauma related, medically complex, and would require a high utilization of medical resources. We hypothesized that barriers to medical care would contribute to many presenting complaints, correlating with the severity of patient presentations [5]. By analyzing medical complexity and the contribution of barriers to health care for medical or psychiatric (med/psych) and trauma-related ED encounters of incarcerated patients, we identified potential opportunities for improvement and cost savings within the correctional healthcare system.

## Materials and methods

### Study design

We conducted a retrospective, observational cohort study by reviewing the electronic medical records of all incarcerated patients evaluated in the adult ED of a single, academic, tertiary care facility between January 2012 and December 2014. We collected data on age, race, gender, diagnosis, disposition, and access to medical care. For patients presenting with trauma-related complaints, we documented the cause, mechanism of injury, and need for procedures and specialty medical consultation. We also performed a comparative analysis on hospital admission rates for incarcerated patients and non-incarcerated patients presenting to the ED at our institution. Because information was previously de-identified and only studied in aggregate, this study qualified for Institutional Review Board exemption.

### Patient population

The study population included incarcerated patients evaluated in the adult ED for medical, psychiatric, and trauma-related complaints. Because psychiatric patients presenting to our facility require medical evaluation, we analyzed psychiatric and medical patients in a single cohort (med/psych). We excluded non-incarcerated patients, patients transferred from other medical facilities including satellite EDs within our hospital system, patients transferred from inpatient psychiatric facilities, patients who initially presented to the children’s ED, and patients who were arrested but not formally incarcerated.

### Data handling and compilation

Data were retrieved from a de-identified database previously generated for an ED quality improvement initiative. Barriers to medical access included any reported event that limited a patient’s access to health care: the discontinuation of prescribed medications, delayed presentation following an injury, or a missed physician appointment. Because reconstruction of the raw data was not possible, we characterized barriers as “not specified” when data indicated that a barrier was present but did not include enough detail to determine the barrier type. Hospital admission rates for non-incarcerated patients were derived from ED data originally collected from January 2012 through December 2014 as part of ongoing, internal quality and safety monitoring study.

### Data analysis

Continuous data were summarized using descriptive statistics and analyzed using an unpaired student’s t-test or one-way analysis of variance. Categorical data were summarized into frequency counts, percentages, and contingency tables, which were analyzed using chi-square analysis or Fisher’s exact test. For the comparative analysis of hospital admission rates with non-incarcerated patients, incarcerated patient ED encounters were subtracted from hospital totals as appropriate. All statistical analyses were calculated using GraphPad Prism 7 software for Macintosh (La Jolla, California).

## Results

**Fig 1:**
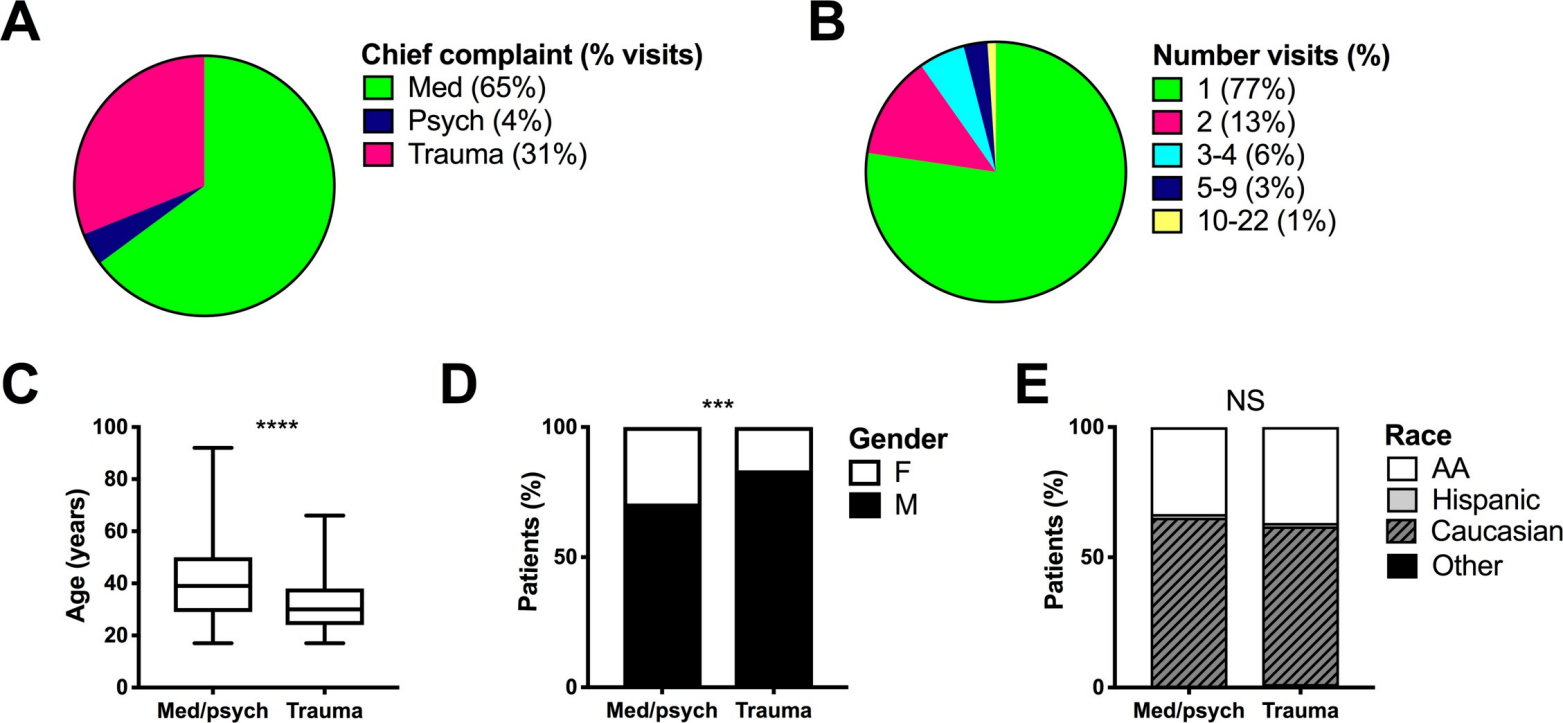
Descriptive data of emergency department visits and patient demographics. The frequency of chief complaint type (A) and the number of visits per patient (B) were calculated for 894 emergency department visits and 574 incarcerated patients, respectively, who presented to a tertiary care, academic emergency department between January 2012 and December 2014. Average age (calculated based on 574 patients; C), gender (D), and race (E) were quantified according to the chief complaint category. Shown in percent (%) of patients unless otherwise indicated. Error bars in (C) denote age range in years. AA, African American; F, female; M, male; med, medical; psych, psychiatric; NS, no statistical difference. **** p < 0.0001, *** p = 0.0005.

We analyzed 894 ED encounters of 574 incarcerated patients who presented to the ED between January 2012 and December 2014. Patient encounters included 580 (64.9%) medical, 36 (4.0%) psychiatric (based on the primary diagnosis), and 278 (31.1%) trauma-related emergencies (Fig 1A). Of 574 patients, 444 (77.4%) patients represented a single ED encounter (Fig 1B). Average patient age was 37 years, with a median of 35 years, and a range of 17 to 92 years. Medical patients were significantly older than psychiatric or trauma patients, with an average patient age of 40.2, 34.7, and 33.0 years respectively (p < 0.0001, medical versus psychiatric: mean difference 5.492 [CI 0.3131–10.67] and medical versus trauma: mean difference 7.219 [CI 5.019–9.418]; S1A Fig). In the state of Tennessee, a mobile team of experts called the Crisis Response Team often evaluates uncomplicated psychiatric cases at the jail for direct admission to inpatient psychiatric facilities, avoiding ED involvement. Thus, patients with isolated psychiatric conditions that do not require medical clearance or stabilization (such as isolated depression, suicidal ideation, or non-acutely dangerous psychosis) are kept in the local jail clinics where they can be evaluated via telemedicine by psychiatric crisis response staff. Therefore, an incarcerated patient with a primary psychiatric complaint will only be transferred to the ED if there is a potential complicating medical condition. Due to this unique situation in our region and the retrospective nature of our study, we analyzed patients with primary medical complaints and patients with psychiatric complaints together (med/psych). In the combined analysis, patients presenting with med/psych complaints remained significantly older than patients presenting with trauma-related complaints (39.9 versus 33, p < 0.0001).

The majority of patient encounters were with white (63.4%) males (75.6%). Gender and race demographics were further characterized based on encounter type (Fig 1D and 1E). A higher frequency of males presented with trauma-related complaints than with med/psych complaints (83.4% versus 70.7%, p = 0.0005). The gender of patients presenting with medical complaints did not differ significantly from patients presenting with psychiatric complaints (S1B Fig). White, African American, and Hispanic patients comprised 65.2%, 33.3%, and 1.4% of med/psych encounters and 60.5%, 36.8%, and 1.3% of trauma encounters, respectively. Race frequencies did not differ significantly based on chief complaint category (Fig 1E and S1C Fig).

**Fig 2:**
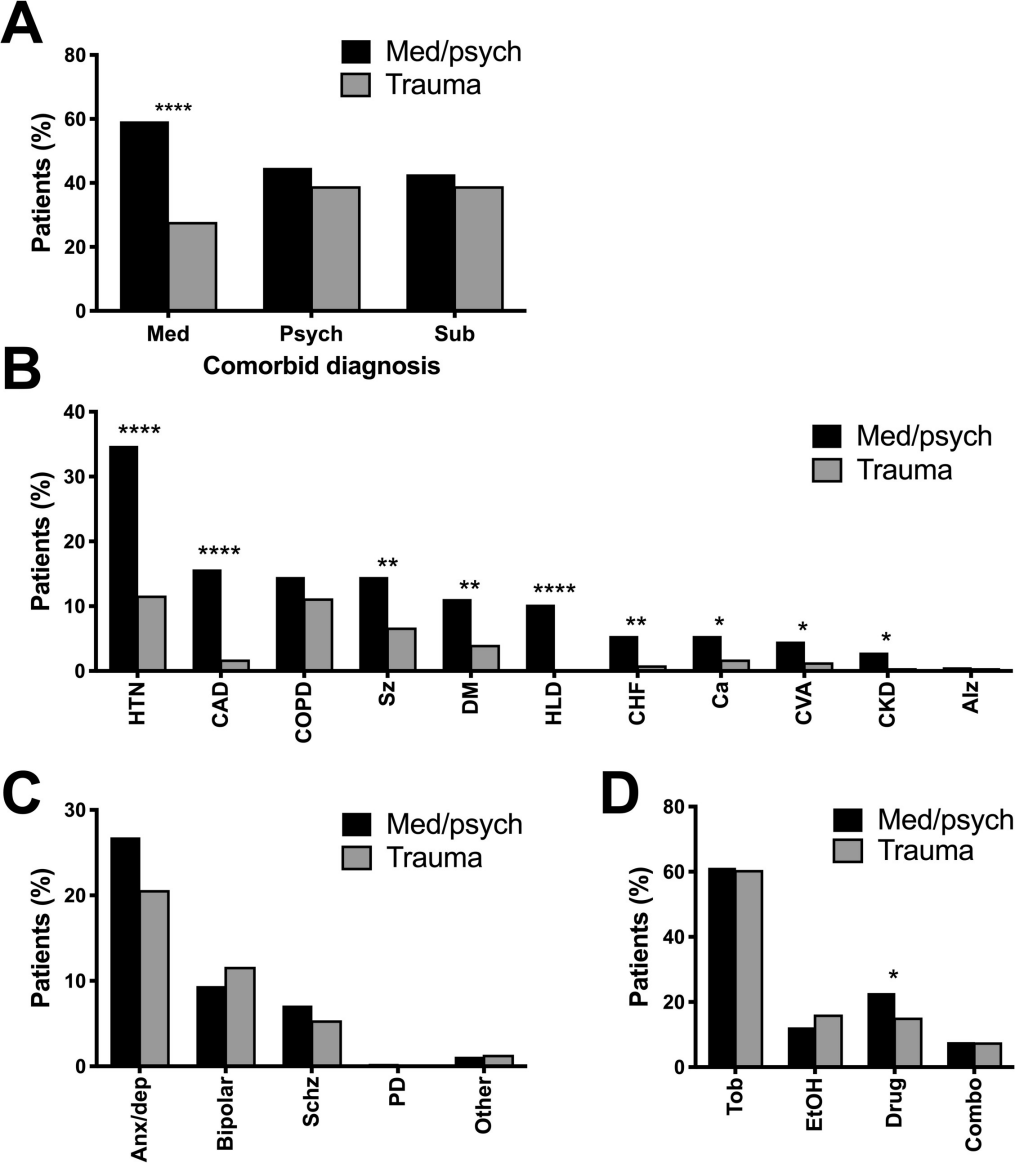
Patients with medical or psychiatric chief complaints had more medical comorbidities than patients with trauma-related complaints. For incarcerated patients presenting to a tertiary care, academic emergency department between January 2012 and December 2014 with medical or psychiatric and trauma-related complaints, the percent of medical, psychiatric, or substance abuse disorder (A) comorbidities was calculated. The percent of individuals with specific medical (B), psychiatric (C), and substance use disorder (D) comorbidities was quantified. Shown in percent (%) of patients. Alz, Alzheimer’s disease or dementia; anx/dep, anxiety and/or depression; Ca, cancer; CAD, coronary artery disease or history of a myocardial infarction; CHF, chronic heart failure; CKD, chronic kidney disease; Combo, combined drugs and alcohol; COPD, chronic obstructive pulmonary disease or asthma; CVA, cerebral vascular accident or history of a transient ischemic attack; DM, diabetes mellitus; EtOH, alcohol; HLD, hyperlipidemia; HTN, hypertension; PD, personality disorder; Psych, psychiatric; Schz, schizophrenia or psychotic disorder; Sub, substance abuse; Sz, seizures; Tob, tobacco. * p < 0.05, ** p ≤ 0.005, **** p < 0.0001.

We observed a higher prevalence of comorbid medical conditions in incarcerated patients presenting with med/psych complaints compared with patients presenting with trauma-related complaints (59.3% versus 27.8%, p < 0.0001, RR 1.638 [CI 1.43–1.88]; Fig 2A). For incarcerated patients presenting with med/psych complaints, the most common medical comorbidities in order of frequency were hypertension (HTN; 34.8%), coronary artery disease or a history of myocardial infarction (15.7%), chronic obstructive pulmonary disease or asthma (14.5%), seizures (14.5%), diabetes mellitus (DM; 11.1%) and hyperlipidemia (10.3%; Fig 2B). Overall, comorbid medical conditions observed in patients presenting with trauma-related complaints were similar in type and distribution and lower in prevalence compared to patients presenting with med/psych complaints (Table 1).

**Table 1:** Medical, psychiatric, and substance use comorbidities in incarcerated patients.

Comorbidity	Med/psych (% patients)	Trauma (% patients)	Relative Risk	Confidence Intervals	p-value
Any medical comorbidity	59.3	27.8	1.64	1.43–1.88	<0.0001
Hypertension	34.8	11.7	1.53	1.36–1.72	< 0.0001
Coronary artery disease or history of myocardial infarction	15.7	1.8	1.62	1.43–1.78	< 0.0001
Chronic obstructive pulmonary disease or asthma	14.5	11.2	1.11	0.92–1.3	0.25
Seizures	14.5	6.7	1.31	1.1–1.49	0.004
Diabetes mellitus	11.1	4.0	1.37	1.34–1.56	0.003
Hyperlipidemia	10.3	0	1.71	1.54–3.66	< 0.0001
Congestive heart failure	5.4	0.9	1.51	1.18–1.68	0.005
Cancer	5.4	1.8	1.37	1.04–1.58	0.03
Cerebrovascular disease or transient ischemic attack	4.6	1.3	1.4	1.03–1.61	0.04
Chronic kidney disease	2.8	0.4	1.5	1.02–1.68	0.041
Alzheimer’s disease or dementia	0.6	0.4	1.09	0.34–1.55	0.84
Any psychiatric comorbidity	44.7	39.0	1.10	0.96–1.25	0.18
Anxiety or depression	26.8	20.6	1.13	0.98–1.30	0.09
Bipolar disorder	9.4	11.7	0.91	0.70–1.11	0.39
Schizophrenia	7.1	5.4	1.11	0.84–1.35	0.41
Personality disorder	0.3	0.0	1.64	0.34–3.50	0.43
Other psychiatric comorbidity	1.1	1.3	0.93	0.41–1.39	0.83
Any substance use disorder	42.7	39.0	1.06	0.93–1.21	0.38
Alcohol use disorder	12.3	16.1	0.87	0.69–1.06	0.19
Illicit substance use disorder	22.8	15.2	1.19	1.02–1.36	0.03
Combination (alcohol and illicit substance use disorder)	7.7	7.6	1.0	0.76–1.23	0.98
Tobacco use disorder	61.3	60.5	1.01	0.89–1.16	0.86

Data shown in % of patients. The category of any substance use disorder does not include tobacco use. med, medical; ns, not significant; psych, psychiatric.

Incarcerated patients presenting with med/psych and trauma-related complaints commonly reported the presence of any psychiatric condition (44.7% and 39%, respectively; Fig 2A and Table 1), anxiety or depression (26.8% and 20.6%, respectively; Fig 2C), and any substance use disorder, which included alcohol and/or drugs but not tobacco (42.7% and 39.0%, respectively; Fig 2A). Nearly two-thirds of inmates reported tobacco use disorder (Fig 2D). In contrast to medical comorbidities, we observed no difference in the prevalence of psychiatric illness (Fig 2A–2C) or the presence of any substance use disorder (Fig 2A) in incarcerated patients presenting with med/psych complaints compared to patients presenting with trauma-related complaints. Of specific substance use disorders surveyed, only drug abuse was noted to be statistically different between incarcerated patients presenting with med/psych complaints and incarcerated patients presenting with trauma-related complaints (22.8% versus 15.2% p = 0.03, RR 1.19 [CI 1.02–1.36]).

**Fig 3:**
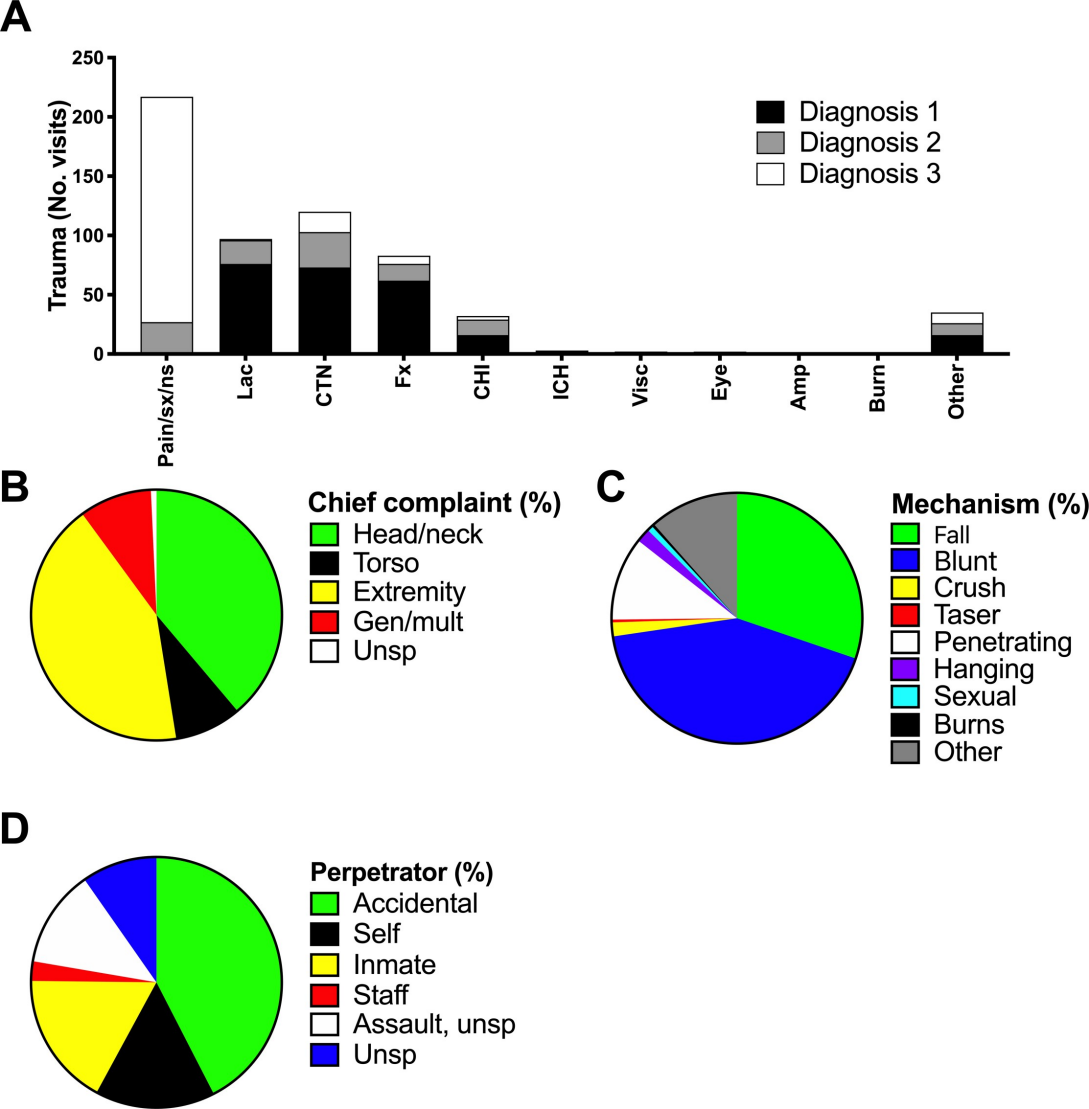
Incarcerated trauma patients present with a range of injuries and mechanisms. Primary, secondary, and tertiary discharge diagnoses (A), primary injury location (B), mechanism of injury (C), and reported perpetrator (D) for 278 trauma-related encounters of incarcerated patients presenting to a tertiary care, academic emergency department between January 2012 and December 2014 were calculated in absolute number (No. visits) or percent (%) of patient encounters as indicated. Amp, amputation; CTN, contusion; Fx, fracture; Gen, general; ICH, intracerebral hemorrhage; Lac, laceration; Multi, multi-organ; Unsp, unspecified; Visc, visceral injury.

Previous studies report that approximately one-third of inmates sustain an injury while incarcerated, half of which are related to violence, and two-thirds are intentional [12,13]. We therefore sought to elucidate the nature and complexity of traumatic complaints in the incarcerated patient population evaluated in our ED. Traumatic injuries were most commonly diagnosed as lacerations (76%), contusions (73%), and fractures (62%; Fig 3A). Injuries most commonly affected an extremity (42.4%) or the head/neck (38.8%; Fig 3B) and resulted from blunt trauma (42.4%) or falls (30.2%; Fig 3C). Only 42.4% of trauma-related complaints were reported as accidental injuries (Fig 3D). Intentional injuries comprised 47.8% of trauma-related ED encounters, with 17.3%, 15.5%, 12.6% and 2.3% attributable to assault by another inmate, self-harm, unspecified assault, or assault by correctional facility staff member, respectively.

**Fig 4:**
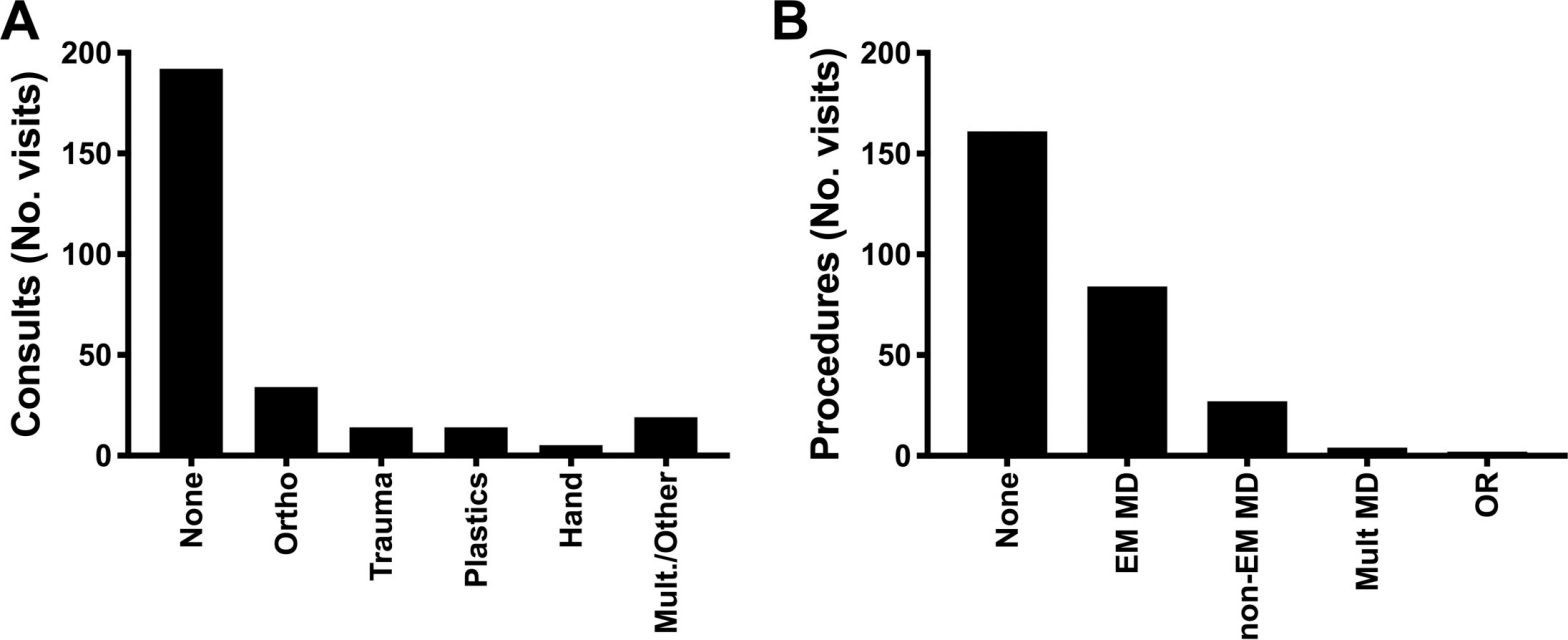
Most trauma-related complaints were adequately managed in the emergency department by emergency medicine physicians. Specialist consultations (A) and procedures (B) required for 278 trauma-related patient encounters for incarcerated patients presenting to a tertiary care, academic emergency department between January 2012 and December 2014 are shown in the number of patient encounters (No. visits). EM, emergency medicine; MD, medical doctor; Mult, multiple; No., number; OR, operating room; Ortho, orthopedics.

**Fig 5:**
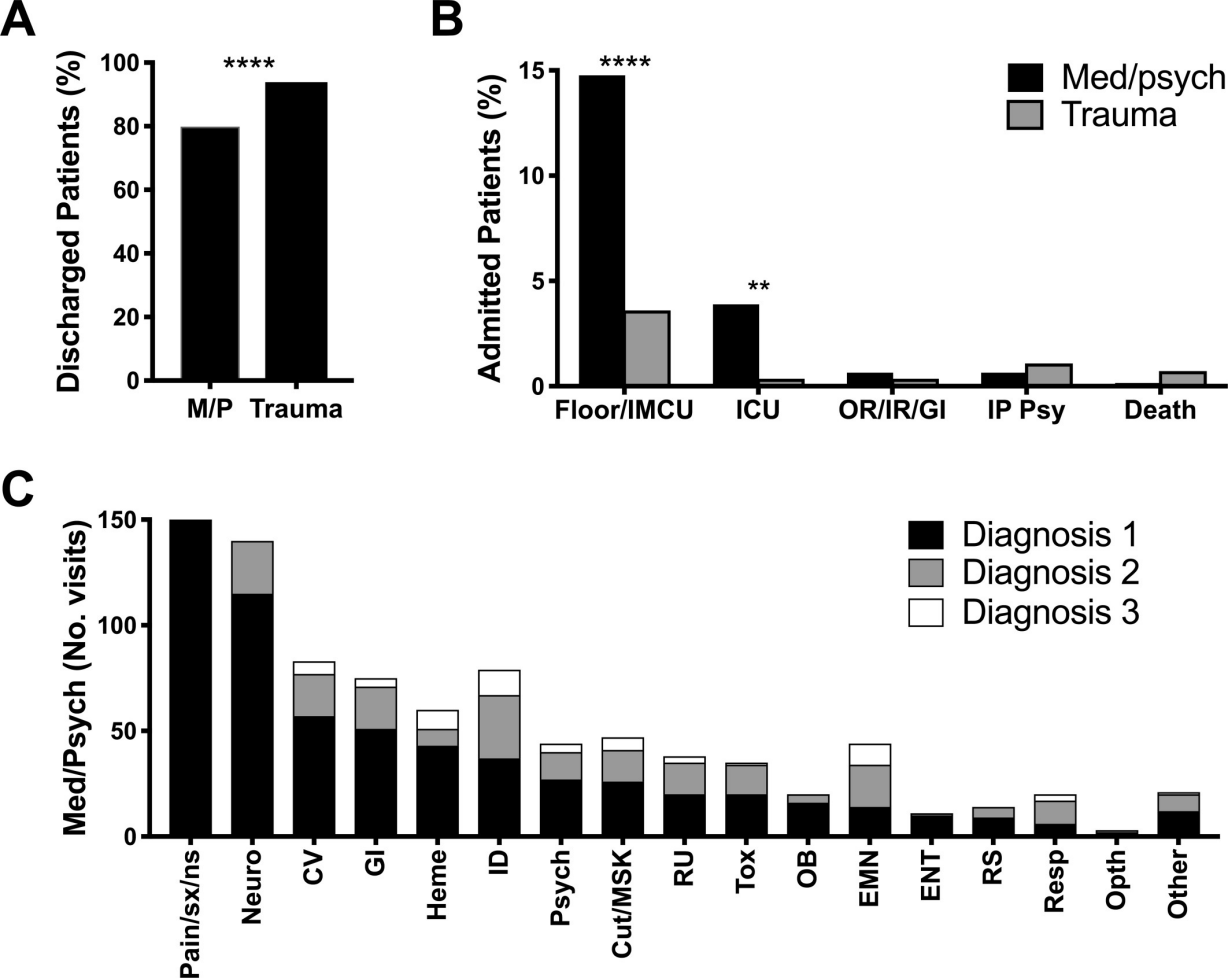
Patients presenting with medical or psychiatric complaints were more likely to require hospital admission than patients presenting with trauma-related complaints. We quantified the percent of incarcerated patients presenting to a tertiary care, academic emergency department between January 2012 and December 2014 who were discharged to the correctional facility of origin (A) or admitted to the hospital (B). Primary, secondary, and tertiary discharge diagnoses were quantified for patients presenting with a medical or psychiatric chief complaint (C). Shown in percent (%) or absolute number (No. visits) of patient encounters as indicated. Cut, cutaneous; CV, cardiovascular; EMN, endocrine/metabolic/nutrition; ENT, ear/nose/throat; GI, gastroenterology; Heme, hematologic; ICU, intensive care unit; ID, infectious disease; IMCU, intermediate medical care unit; IP Psy, inpatient psychiatric facility; IR, interventional radiology; M/P, medical or psychiatric chief complaint; Med, medical; MSK, musculoskeletal; Neuro, neurologic; OB, obstetric; Opth, ophthalmology; OR, operating room; Psych, psychiatric; Resp, respiratory; RS, reproductive system; RU, renal/urinary; Tox, toxicity or intoxication. ** p = 0.003, **** p < 0.0001.

To estimate trauma complexity, we evaluated medical resource utilization, represented by medical specialty consultations and procedures required during each patient encounter. Most trauma patients did not require additional medical expertise beyond that of the emergency medicine (EM) physician (69.1%; Fig 4A). Of the patients who did require a specialty consultation, orthopedic surgery was most frequently consulted (12.2%). Most trauma-related patient encounters required either no procedures (57.9%) or procedures adequately managed by EM physicians (30.2%; Fig 4B). In addition, patients presenting with trauma-related complaints were more likely to be discharged to the correctional facility of origin than patients presenting with med/psych complaints (93.9% versus 79.9%, p < 0.0001, RR 2.88 [CI 1.86–4.57]; Fig 5A).

We next determined the likelihood of hospital admission for incarcerated patients presenting with med/psych complaints compared to incarcerated patients presenting with trauma-related complaints and to non-incarcerated patients evaluated in our ED during the time of the study. Incarcerated patients presenting with med/psych complaints were less likely to be discharged to the correctional facility of origin (Fig 5A) and more likely to require hospital admission either to the floor or intermediate care unit (IMCU; 14.8% versus 3.6%, p < 0.0001, RR 1.36 [CI 1.23–1.47]) or to the intensive care unit (ICU; 3.9% versus 0.4%, p = 0.003, RR 1.41 [CI 1.18–1.5]; Fig 5B) than incarcerated patients presenting with trauma-related complaints. The rate of discharge to the correctional facility of origin for patients presenting with psychiatric chief complaints was not statistically different from that of patients presenting with medical complaints (S2A Fig).

We also compared hospital admissions for the incarcerated patient population presenting to the ED to the non-incarcerated patient population. Of 158,620 non-incarcerated patients presenting to the ED between January 2012 and December 2014, there were 45,551 hospital admissions and 9,698 ICU admissions. Based on these data, incarcerated patients presenting to the ED with med/psych complaints were less likely to require hospital admission (19.2% versus 28.9%, p < 0.0001, RR 0.58 [CI 0.48–0.71]) or ICU admission (3.9% versus 6.2%, p = 0.02, RR 0.62 [CI 0.41–0.93]) than non-incarcerated patients. Categories of discharge diagnoses for patients presenting with med/psych complaints are depicted in Fig 5C.

**Fig 6:**
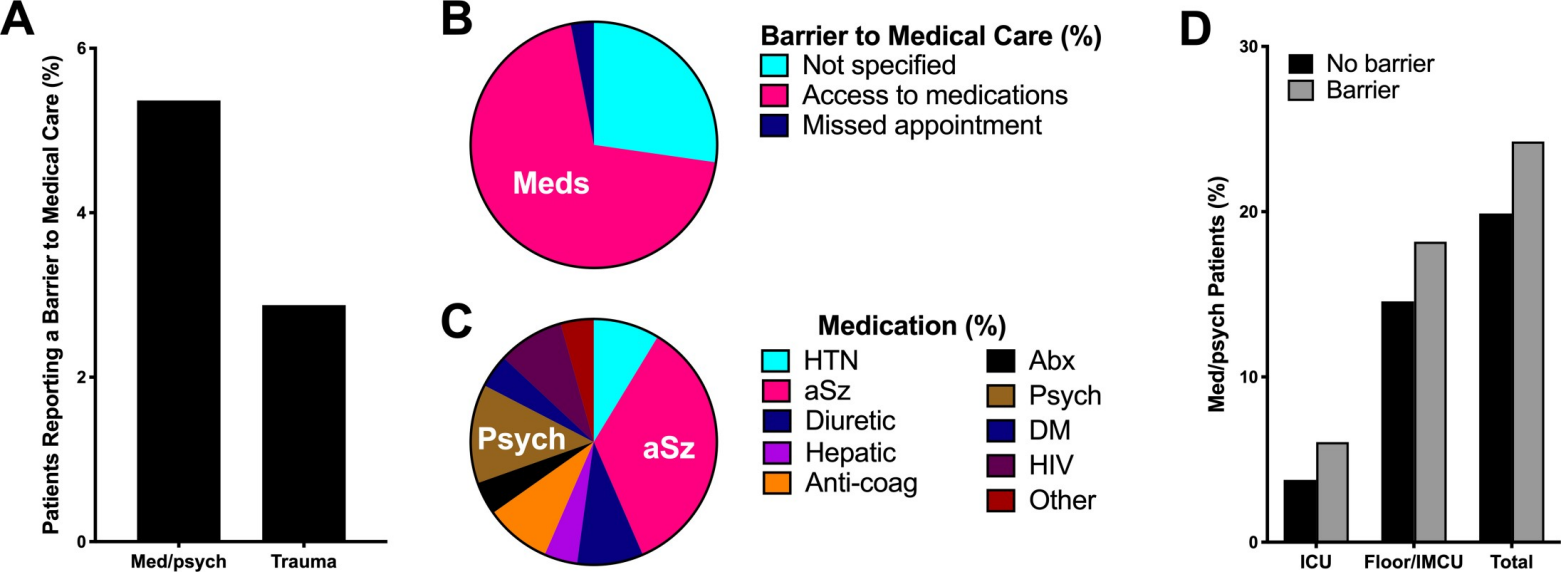
Incarcerated patients report barriers to medical care as contributing to emergency department presentations. The frequency of reported barriers to access to medical care among patients presenting with medical or psychiatric and trauma-related chief complaints was quantified for incarcerated patients presenting to a tertiary care, academic emergency department between January 2012 and December 2014 (A; p = 0.10). The type of barrier to medical care (B) and the category of medication for patients reporting a lack of access to prescription medications (C) was calculated for incarcerated patients presenting with medical or psychiatric chief complaints. We calculated the rate of admission to the intensive care unit, the rate of admission to the floor or the intermediate care unit, and the overall rate of patients who were unable to be discharged to the correctional facility of origin from the emergency department (total) for patients presenting with a medical or psychiatric chief complaint who reported a barrier to medical access as contributing to the emergency department encounter to those who did not report a barrier (D; p > 0.3 for all comparisons). Patients who were unable to be discharged to the correctional facility of origin from the emergency department (total) include patients who required hospital admission, a specialty procedure (gastroenterologic, interventional radiologic, or surgical), transfer to an inpatient psychiatric facility, or patients who expired. Data shown as the percent (%) emergency department encounters. Abx, antibiotics; Anti-coag, anticoagulant; aSz, anti-epileptic; DM, diabetes mellitus; HIV, human immunodeficiency virus; ICU, intensive care unit; IMCU, intermediate care unit; Med, medical; Meds, prescription medications; Psych, psychiatric.

Given the prevalence of barriers to accessing medical care in the correctional healthcare system [9], we explored whether barriers to medical care contributed to the presentation severity of incarcerated patients evaluated in our ED. Patients reported a barrier to medical care in 5.4% of ED encounters related to med/psych complaints and 2.9% of ED encounters related to trauma complaints (p = 0.10; Fig 6A). For patients presenting with a med/psych complaint and citing a barrier to medical care, 69.7% reported a lack of access to prescription medications (Fig 6B). Commonly cited prescription medications included anti-epileptic (34.8%), psychiatric (13%), anti-hypertensive (8.7%), human immunodeficiency virus (8.7%), diuretic (8.7%), and anticoagulant (8.7%) medications (Fig 6C). In contrast, all patients, except one, who presented with trauma-related complaints and indicated the presence of a barrier to medical care reported a delay in medical care as the barrier contributing to the ED presentation (data not shown). The single patient who did not cite a delay of care was unable to be seen by the on-site physician because of incomplete paperwork. For incarcerated patients who reported a barrier to medical care, 75.8**%** of patients presenting with med/psych complaints (Fig 6D) and 100% of patients presenting with trauma-related complaints (data not shown) were discharged from the ED to the correctional facility of origin. Of patients reporting a barrier to medical care and requiring hospital admission, 6.1% required admission to the ICU and 18.2% were admitted to a hospital ward or the IMCU (Fig 6D). The presence of a barrier to medical care was not correlated with patient discharge to the correctional facility of origin (p = 0.51), admission to the hospital floor/IMCU (p = 0.51), or admission to the ICU (p = 0.57). For patients presenting with trauma-related complaints, the presence of a barrier to medical care was not associated with the requirement for a procedure (p = 0.65) or medical specialty consultation (p = 0.24; data not shown).

## Discussion

In the US, the approximate incidence of imprisonment over an individual’s lifetime is 6.6% [14], yet challenges intrinsic to studying incarcerated individuals have resulted in a relative dearth of research on this population [10,11]. Located in Tennessee (TN), which ranks 10^th^ in the nation for its incarceration rate [2], our ED regularly caters to the incarcerated patient population. The majority of these patients present from local jails. In this study, we sought to characterize the local incarcerated patient population, to understand the medical complexity of presenting complaints, to estimate the prevalence of barriers to medical care, and to determine whether these barriers may contribute to the severity of patient presentations.

### Demographics

Incarcerated patients presenting to our ED comprise a heterogeneous cohort of individuals with medical, psychiatric, and trauma-related complaints. Demographically, these patients largely consist of middle-aged, white males. The racial distribution of the incarcerated patient population in this study more closely approximates that observed in TN jails, with an increased frequency of white individuals and a decreased frequency of Hispanic individuals compared with jailed persons nationwide [7,15]. Compared to the local non-incarcerated population, a disproportionate number of males and African Americans are present in the incarcerated patient population, likely reflecting the overrepresentation of males and African Americans in US correctional facilities [16,17]. Interestingly, we observe a slightly higher proportion of female patients in our study’s cohort than the percentage of females in jails in either TN or the US [7]. It is unclear whether this observation reflects a sicker female demographic or an increased likelihood for female inmates to be transported for medical evaluation than their male counterparts. Nonetheless, our observations generally support a population representative of regional jails presenting to our ED.

### Comorbidities

Our descriptive analysis supports previous reports describing the disproportionate rate of medical, psychiatric, and substance use disorders in the incarcerated population [4,5,8]. Compared to non-incarcerated patients presenting to EDs in the US, incarcerated patients presenting to our ED with med/psych complaints report more medical comorbidities, with the exception of DM [18]. Interestingly, the prevalence of HTN in incarcerated patients presenting with med/psych complaints in this study is similar to the rate of HTN reported locally and statewide [16]. Because we lack additional data on the local prevalence of chronic medical conditions, it is difficult to determine whether the higher rate of medical comorbidities observed in our study population reflects a higher rate of local morbidity rather than an increased prevalence of chronic medical conditions in the incarcerated patient population. The incarcerated patient population we evaluated presenting with med/psych complaints reported similar or increased rates of medical comorbidities compared to that reported in the general United States incarcerated population [6,9]. Higher rates of medical comorbidities observed in incarcerated patients presenting with med/psych complaints may reflect a higher frequency of medical visits required by persons with chronic health conditions.

Previous reports estimate that over a third of incarcerated persons suffer from mental health disorders [8], and approximately half meet the definition of drug abuse or dependence [19]. We also observe a higher prevalence of mental illness and tobacco use disorder in the incarcerated patient population compared to the general US population [4,20,21], and a higher prevalence of anxiety or depression, alcohol use disorder, and illicit substance abuse compared to the ED-evaluated population nationwide [18]. The prevalence of anxiety or depression in the incarcerated patient population evaluated in our ED is also higher than that reported in the local population [16]. Compared to incarcerated individuals in the US, we observe similar rates of anxiety or depression, but higher rates of bipolar disorder and schizophrenia [20]. These data support the need for ongoing efforts to address mental illness, substance abuse and smoking cessation in the correctional system [22].

### Medical complexity

Similar to previous research, we observe that incarcerated patients presenting with medical complaints comprise an older cohort than patients presenting with non-medical complaints [23]. Furthermore, patients presenting with med/psych complaints are more likely to have medical comorbidities and require hospital admission than patients presenting with trauma-related complaints, suggesting a higher level of medical complexity in this cohort. Approximately 1 in 5 incarcerated patients presenting to our ED with a med/psych complaint requires hospital admission, and approximately 1 in 5 admitted patients requires ICU care. These admission rates greatly exceed the 2012 nationally reported hospital (11.1%) and ICU (1.4%) admission rates from the ED nationwide [24]. Compared with non-incarcerated patients presenting to our ED, however, we observe a lower admission rate in incarcerated patients presenting with med/psych complaints. Given that our institution is a tertiary care, academic center, level 1 trauma center, stroke center, and a cardiac reperfusion center, we suspect that the high admission rate of nonincarcerated patients reflects the high level of medical complexity of patients evaluated in our ED. Importantly, patient transfers were excluded from the study population, but not from institutional data. Alternatively, it is possible that the higher admission rates in our study and at our institution may reflect local practice standards or a high level of local medical complexity. Nonetheless, our data suggest that the incarcerated patient population presenting with med/psych complaints comprises a more medically complex cohort than the ambulatory patient population presenting to EDs nationwide [18].

### Trauma-related complaints

In our study, trauma-related complaints account for 31% of all ED encounters, which is slightly higher than the 28.3% quantified in the ED nationally [18]. Given the high incidence of traumatic injury in imprisoned populations [12,13], it is surprising that the proportion of trauma-related incidents in this study’s cohort approximates that observed in EDs nationwide. Of note, studies indicate that the number of traumatic injuries may be underreported [25].

Typical mechanisms of injury in this study include falls and blunt trauma, with the majority of traumatic injuries affecting the head/neck or extremities, similar to that observed for trauma-related complaints presenting to US EDs [24]. Nationally, 72.5% of trauma-related ED complaints are reported as accidental, while only 6.0% are intentional [18]. In comparison, almost half of trauma-related complaints in this study are intentional and include injuries resulting from assault and self-harm. These data are in agreement with previous reports, including the work of Ludwig, *et al*. and Siegler, *et al*. that depict high rates of intentional trauma and violence among inmates [12,13,25]. High rates of intentional injury indicate a role for programs promoting safety in local correctional facilities [13]. While our data are in agreement with those published by others, it should be noted that nationally, the rate of possible false reporting of assaults as “slip and fall” injuries has been pointed out as a source of concern [13]. Our data show an equally high rate of these reported injuries highlighting a possible need for increased diligence in providing a safe and private space for history taking and examination.

Compared to patients presenting with med/psych emergencies, patients presenting with trauma-related complaints are younger [13,23], have fewer medical comorbidities, and are more likely to be discharged to the correctional facility of origin. The majority of incarcerated patients’ injuries were adequately managed by the EM physician and did not require additional hospital resources beyond that available in the ED. These data indicate a potential role for the outpatient management of injuries occurring in incarcerated patients. Expanding programs already in place, such as telemedicine, could result in substantial cost savings [26,27].

### Barriers to health care access

Data published by Wilper, *et al*. indicate that a significant portion of incarcerated individuals fail to receive prescribed medication, medical examinations and blood tests while incarcerated [9]. In our study, a small percent of incarcerated patients evaluated in this study report barriers to medical care as contributing to the ED presentation. Our data was extremely limited as this was a chart review for quality analysis and only those charts in which the provider noted a specific barrier to care, such as “Patient states he has not been given his usual seizure medicine while in jail.” Given that we noted a significant number of charts that recorded barriers, this likely represents the “tip of the iceberg” and signifies a need for more investigation into barriers in our system. We observe a trend toward an increased frequency of barriers to medical care in incarcerated patients presenting with med/psych complaints compared to patients presenting with trauma-related complaints. Notably, the barriers cited are different between the two patient groups. Patients presenting with med/psych complaints most commonly report lack of access to prescription medications, whereas patients presenting with trauma-related complaints cite delays in medical care. Delays in assuming care can lead to increased complexity of the presenting complaint [28]. However, all patients citing a delay in medical care were discharged to the correctional facility of origin from the ED, indicating minimal impact on injury complexity. It should be noted that in this study, we were unable to analyze the effectiveness of follow up when patients were referred for follow up at ED discharge. Inquiries to the medical clinics at the jails involved in this study revealed that polices are in place to safeguard patient adherence to follow up appointments, but there no data available describing the actual rates of follow up.

This project was conducted as a quality review in our institution. As such, it became apparent that our institution lacks sufficient policies to ensure proper care of incarcerated patients. Opportunities for improved care included physician education regarding the need for these patients to be evaluated privately, without the presence of correctional facility staff, as well as education on capabilities available at regional jails (such as what medications are available in the onsite clinics). This educational information has now been integrated into the standard educational curriculum for Emergency Medicine Resident Physicians. Ongoing opportunities and efforts include improving communication and coordination of follow up care, as well as advocating for the timely evaluation by medical staff of new inmates and maintenance of home medication regimens.

Given the increased burden of chronic medical conditions observed in patients presenting with med/psych complaints, and the increased complexity of med/psych complaints compared with trauma-related complaints, we suspected that patients presenting with med/psych complaints would be especially vulnerable to interruptions in their medical care. However, we observe no association between the overall, floor/IMCU, or ICU admission rate and cited barriers to medical care. For trauma-related complaints, the delay in medical care is not associated with an increased requirement of hospital resources. These results suggest that the presentation severity for patients reporting a barrier to medical care is similar to that for patients who do not report a barrier to medical care. Importantly, we only included a barrier to medical care in this analysis if it was specifically documented in the patient’s chart. Therefore, it is feasible that the prevalence of barriers to medical care is underestimated in this study [9] and that a more comprehensive analysis might reveal an association between barriers to medical care and medical complexity. Nonetheless, the presence of identifiable barriers to medical care indicates opportunities to better manage the chronic health conditions of incarcerated individuals.

### Limitations

Given the retrospective nature of this research, our study serves to generate hypotheses for future study. Variation in medical staff documentation, errors in patient self-reporting, and potential confounding variables for which we lacked data, including the patient’s sentence length, whether a patient’s presentation influenced the chosen medical facility, variability in the medical care available at each patient’s correctional facility, and the day of the week of presentation (some jails have weekday clinics), may have influenced our results. Because this was a small, single center study, our analyses may lack generalizability to a larger population. Serving as the receiving facility for a vast number of medically complex cases contributes to the high acuity of complaints evaluated in our ED. We were unable to control for potential confounding variables in comparisons made with the non-incarcerated population for which we lacked data, such as age and information on patients transferred from other medical facilities.

## Conclusions

Incarcerated patients comprise a vulnerable population: detained individuals who are often susceptible to lapses in medical care because of chronic health comorbidities, mental disparities, and substance use disorders. While we observe no association between barriers to medical care and medical complexity, barriers were likely underestimated in this study. Opportunities for healthcare improvement include promoting the continuity of health care for incarcerated patients, ensuring access to prescription medications, and providing alternate accommodations for missed medical appointments. Additional health issues could be addressed through the expansion of programs that provide access to mental health resources and substance abuse treatment, as well as programs aimed to prevent violence and injury [13,19,29]. While the expeditious evaluation of trauma victims remains important, costs related to traumatic injuries may be mitigated through outpatient management. In addition, policymakers should consider the fact that many hospitals are unable to receive compensation for the emergency care of these patients as Medicare usually only provides reimbursement for stays of longer than 24 hours. Research evaluating such interventions would result in enhancing community health and allocating resources more effectively. Given the high turnover of jails, correctional facilities are in the unique position to act as an extension of the public health institution, thereby directing resources to one of society’s most vulnerable populations [4,30].

## Supporting information

S1 Fig Descriptive data of emergency department visits and patient demographics. Average age (A), gender (B), and race (C) were quantified according to chief complaint category for 574 incarcerated patients presenting with medical, psychiatric, and trauma-related chief complaints to a tertiary care, academic emergency department between January 2012 and December 2014. Shown in percent (%) patients unless otherwise indicated. Error bars in (A) denote age range in years. AA, African American; F, female; M, male; med, medical; psych, psychiatric; For A, p < 0.0001. * mean difference 5.492 (CI 0.3131–10.67) **** mean difference 7.219 (CI 5.019–9.418).
(TIF)

S2 Fig Patients presenting with a medical or psychiatric chief complaint were more likely to be admitted to the hospital than patients presenting with a trauma-related chief complaint. Disposition of patients to the correctional facility of origin (A) or admission to the hospital (B) for all emergency department encounters with incarcerated patients presenting to a tertiary care, academic emergency department between January 2012 and December 2014. Shown in the percent (%) or absolute number (No. visits) of patient encounters, as indicated. GI, gastroenterology; ICU, intensive care unit; IMCU, intermediate care unit; IP Psy, inpatient psychiatric facility; IR, interventional radiology; Med, medical; OR, operating room; Psych, psychiatric.
(TIF)

S1 File (XLSX)
