# Supplementary figures and images for: Emergency medical care of incarcerated patients: Opportunities for improvement and cost savings
Source: PLoS One. 2020 Apr 27;15(4):e0232243. doi: 10.1371/journal.pone.0232243 (PMC7185724; doi:10.1371/journal.pone.0232243)

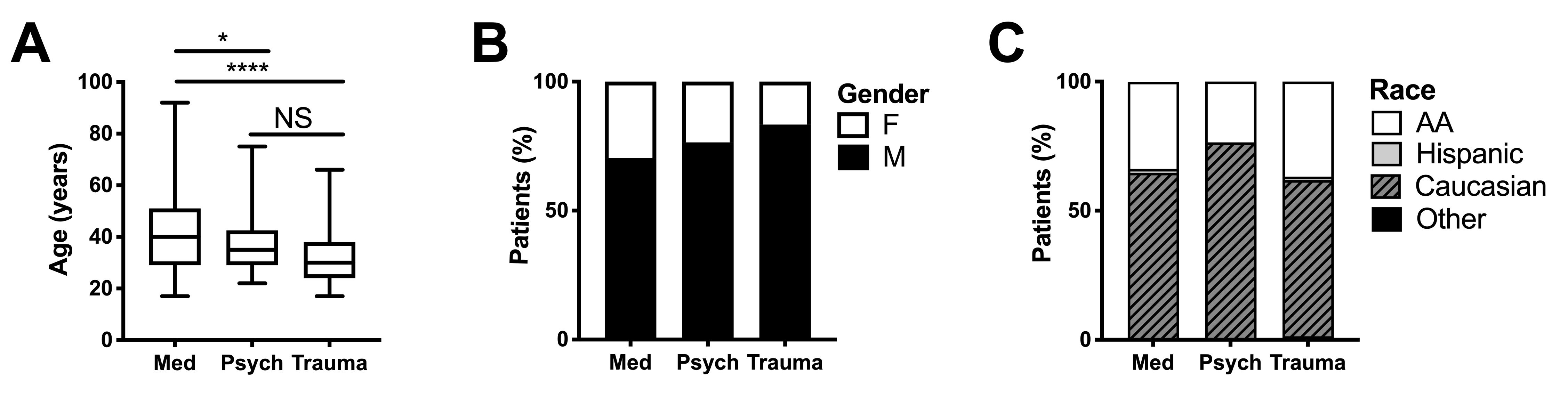

Supplement: S1 Fig — Average age (A), gender (B), and race (C) were quantified according to chief complaint category for 574 incarcerated patients presenting with medical, psychiatric, and trauma-related chief complaints to a tertiary care, academic emergency department between January 2012 and December 2014. Shown in percent (%) patients unless otherwise indicated. Error bars in (A) denote age range in years. AA, African American; F, female; M, male; med, medical; psych, psychiatric; For A, p < 0.0001. * mean difference 5.492 (CI 0.3131–10.67) **** mean difference 7.219 (CI 5.019–9.418). (TIF) [file pone.0232243.s001.tif]

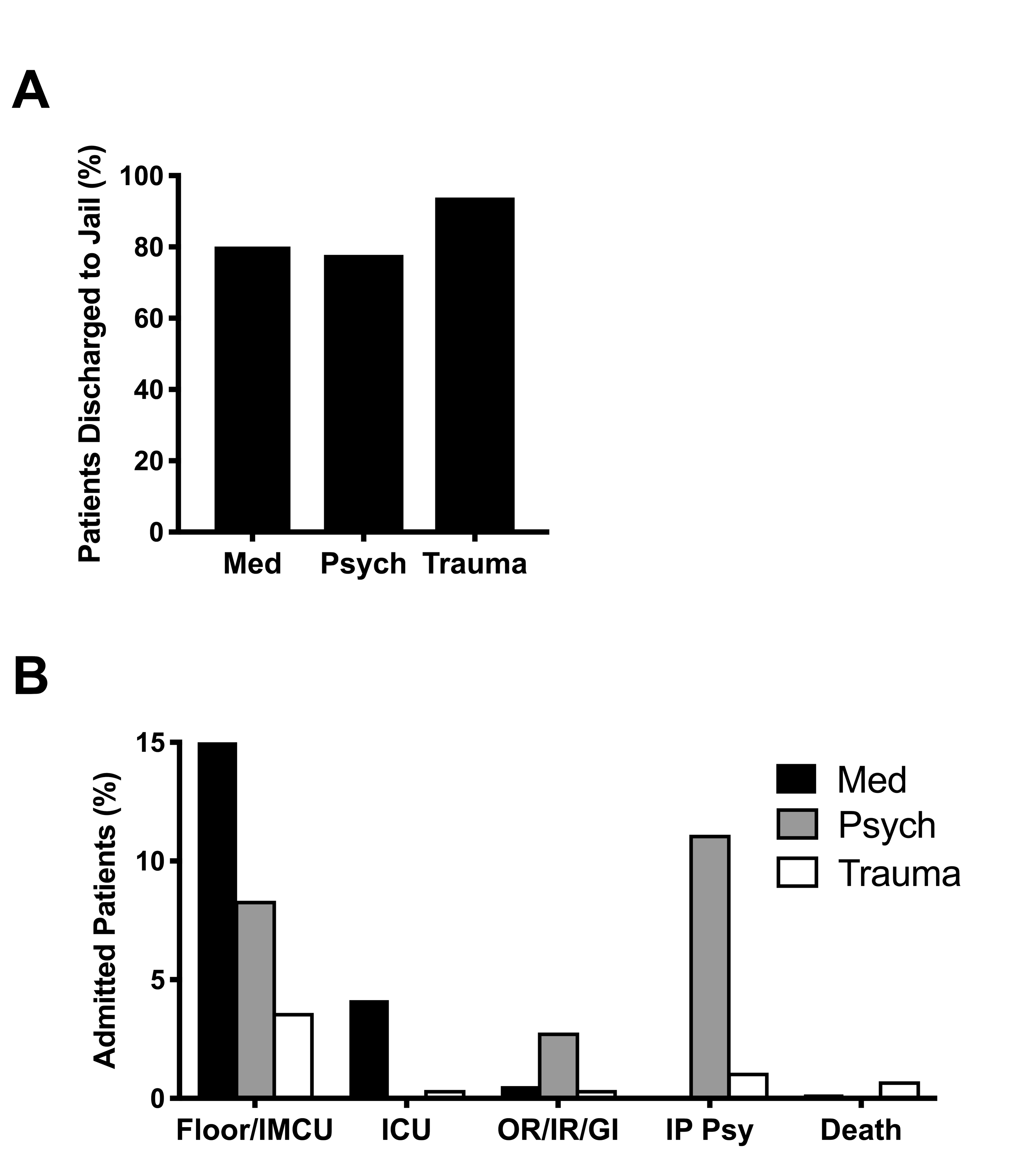

Supplement: S2 Fig — Disposition of patients to the correctional facility of origin (A) or admission to the hospital (B) for all emergency department encounters with incarcerated patients presenting to a tertiary care, academic emergency department between January 2012 and December 2014. Shown in the percent (%) or absolute number (No. visits) of patient encounters, as indicated. GI, gastroenterology; ICU, intensive care unit; IMCU, intermediate care unit; IP Psy, inpatient psychiatric facility; IR, interventional radiology; Med, medical; OR, operating room; Psych, psychiatric. (TIF) [file pone.0232243.s002.tif]
